# *Bactericera cockerelli* resistance in the wild tomato *Solanum habrochaites* is polygenic and influenced by the presence of *Candidatus* Liberibacter solanacearum

**DOI:** 10.1038/s41598-019-50379-7

**Published:** 2019-10-01

**Authors:** Carlos A. Avila, Thiago G. Marconi, Zenaida Viloria, Julianna Kurpis, Sonia Y. Del Rio

**Affiliations:** 1 Texas A&M AgriLife Research and Extension Center, Weslaco, TX 78596 USA; 2 Department of Horticultural Sciences, Texas A&M University, College Station, TX 77843 USA; 3 Present Address: University of Kentucky Research and Education Center, Department of Entomology, 1205 Hopkinsville St., Princeton, KY 42445 USA; 4 Present Address: University of Texas- Rio Grande Valley, Edinburg, TX 78539 USA

**Keywords:** Quantitative trait loci, Plant breeding, Microbe

## Abstract

The tomato-potato psyllid (TPP), *Bactericera cockerelli*, is a vector for the phloem-limited bacterium *Candidatus* Liberibacter solanacearum (Lso), the causative agent of economically important diseases including tomato vein-greening and potato zebra chip. Here, we screened 11 wild tomato relatives for TPP resistance as potential resources for tomato (*Solanum lycopersicum*) cultivar development. Six accessions with strong TPP resistance (survival <10%) were identified within *S. habrochaites*, *S. pennelli*, *S. huaylasense*, *S. chmielewskii*, *S. corneliomulleri*, and *S. galapagense*. Two *S. pennelli* and *S. corneliomulleri* accessions also showed resistance to Lso. We evaluated recombinant inbred lines (RILs) carrying resistance from *S. habrochaites* accession LA1777 in the *S. lycopersicum* background and identified major quantitative trait loci (QTLs) responsible for adult TPP mortality and fecundity in several RILs carrying insertions in different chromosomes, indicating the polygenic nature of these traits. Analysis of a major resistance QTL in RIL LA3952 on chromosome 8 revealed that the presence of Lso is required to increase adult TPP mortality. By contrast, the reduced TPP oviposition trait in LA3952 is independent of Lso. Therefore, resistance traits are available in wild-tomato species, although their complex inheritance and modes of action require further characterisation to optimise their utilisation for tomato improvement.

## Introduction

The tomato-potato psyllid (TPP), *Bactericera cockerelli* (Sulc) (Homoptera: Psyllidae), is a vector for the phloem-limited bacterium *Candidatus* Liberibacter solanacearum (Lso) (syn. *Ca*. L. psyllarous), the causative agent of economically important plant diseases in solanaceous crops including zebra chip in potato (*Solanum tuberosum*) and vein-greening in tomato (*Solanum lycopersicum*)^1–3^. Vein-greening symptoms include chlorosis, leaf mottling, midvein curling, and stunted growth, resulting in small, poor-quality fruit, which drastically reduces their economic value^4,5^. In addition to transmitting Lso, direct TPP feeding damage is a serious problem in tomato production, since TPP can inject toxins into the plant vascular system, resulting in a condition known as ‘psyllid yellows’^6^. Symptoms of TPP infestation include stunted growth, chlorosis or reddening of leaves, and yield reductions, resulting in reported losses of up to 85% in commercial fields^3,7^.

In the absence of resistant cultivars against both the vector and pathogen, growers have to rely on pesticide use to control the vector and therefore reduce pathogen transmission^1,8^. However, the effectiveness of insecticides differs among tomato cultivars^6^ and has drastically decreased due the recent appearance of pesticide-resistant psyllid populations^9^. Natural enemies of TPP can be used as part of an integrated pest management strategy. Several natural enemies have been identified, including minute pirate bug (*Orius tristicolor*), western big-eyed bug (*Geocoris pallens*), and convergent lady beetle (*Hippodamia convergens*)^10^, but they might not provide enough control to protect tomato fields during outbreaks. Plant host resistance represents a more effective approach to sustainably controlling this vector-pathogen complex: the higher the genetic plant baseline, the lower the managerial input required for production^11^. Therefore, identifying sources of genetic resistance is the first step towards developing cultivars with improved performance in the field.

Modern tomatoes exhibit narrow genetic diversity: <5% of the total genetic variation present in the wild species and primitive varieties is present in commercial tomato cultivars^12^. Consequently, modern tomatoes may taste good and look attractive, but they lack many genes that allow them to withstand pests, diseases, and biotic stresses^13,14^. This reality comes in the backdrop of the rich genetic pool of the Solanaceae family, which includes more than 3,000 species with diverse origins spread across vast geographical regions^14^. Tomato belongs to the *Solanum* sect. lycopersicon, a relatively small clade within the Solanaceae family consisting of 14 species or subspecies including the cultivated tomato, *S. lycopersicum*. The natural habitat of tomato species encompasses diverse climatic, geographic, and environmental regions ranging from arid zones to tropical rainforests, which greatly contributes to the vast genetic variation within the clade^15^. However, the development of new cultivars with enhanced resistance or tolerance has been hindered by the lack of genetic diversity within cultivated *S. lycopersicum* germplasm due to its domestication and breeding^16^. Fortunately, germplasm collections of related wild species with broad morphological, physiological, and metabolic diversity are available and have served as the main source of agronomic trait introgression over the past 80 years, including pest and disease resistance, fruit quality, and abiotic stress tolerance^16^. Examples of important introgressed traits include chill tolerance from *S. habrochaites*^17^, late blight resistance from *S. pimpinellifolium*^18^, *Tomato yellow leaf curl virus* (TYLCV) resistance from several species^19–21^, tomato spotted wilt virus resistance from *S. peruvianum*^22^, and fruit flavour-related volatiles from *S. pimpinellifolium*^23^.

One of the best-studied cases of resistance introgression is the the *Mi-1.2* gene from the wild-relative species *S. peruvianum*^24^, which confers resistance to certain root knot nematode species (*Meloidogyne* spp.)^25^, sweet potato whitefly (*Bemisia tabaci*)^26^, and various biotypes of potato aphid (*Macrosiphum euphorbiae*)^27^. In addition, this gene negatively influences TPP host preference and reduces pest fecundity by approximately 50% in tomato^8,28^. Unfortunately, these levels of resistance might not be sufficient to reduce the economic impact of psyllid infestations and its transmitted bacteria in commercial fields. Furthermore, Mi-1.2 is destroyed by heat stress, reducing its effectiveness for conferring resistance to whiteflies and nematodes^29^, thereby diminishing its utility in areas where high temperatures are present. In addition to *S. peruvianum*, resistance to TPP has been detected in another wild relative, *S. habrochaites*, representing a potential source of resistance^30^ for use in breeding programs.

The aims of the current study were to (1) identify potential sources of resistance to TPP and Lso in wild tomato species; and (2) characterise TPP resistance in *S. habrochaites* and its interaction with Lso, the pathogen carried by TPP. We identified six wild tomato accessions with resistance to TPP, two of which also showed resistance to Lso. We characterised resistance in *Solanum habrochaites* accession LA1777 by taking advantage of the availability of mapped recombinant inbred lines (RILs). Major quantitative trait loci (QTLs) involved in adult insect mortality and fecundity were identified on different chromosomes, indicating that resistance in *S. habrochaites* is polygenic, with each QTL controlling different aspects of this trait. Furthermore, by performing functional analysis of *S. habrochaites* RIL LA3952 carrying a major QTL on chromosome 8, we determined that salicylic acid (SA) signalling is enhanced in response to TPP infestation without the presence of its transmitted bacteria, Lso, which is contrary to what was observed in susceptible plants. Finally, we discuss the utility of *S. habrochaites* in tomato breeding in light of the complex inheritance of the TPP resistance trait and the potential for genetic barriers for trait introgression.

## Results and Discussion

### Resistance to TPP and Lso is present in wild tomato relative species

**Figure 1:**
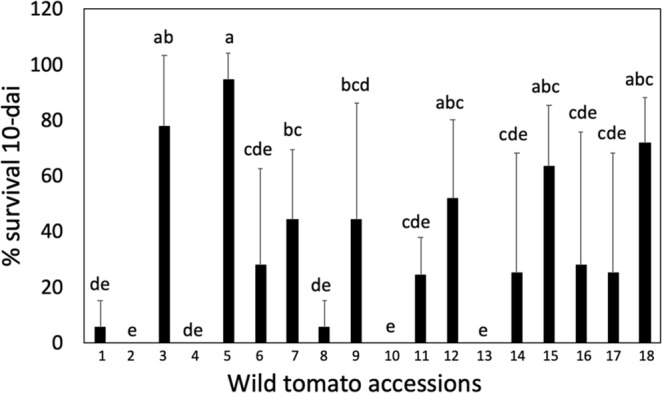
Tomato-potato psyllid survival on wild tomato species at 10 days after infestation (dai). (1) *S. galapagense* (LA0528), (2) *S. pennellii* (LA0716), (3) *S. pimpinellifolium* (LA0722), (4) *S. chmielewskii* (LA1028), (5) *S. cheesmaniae* (LA1037), (6) *S. habrochaites* (LA1223), (7) *S. corneliomulleri* (LA1274), (8) *S. corneliomulleri* (LA1293), (9) *S. neorickii* (LA1326), (10) *S. habrochaites* (LA1777), (11) *S. pennellii* (LA1926), (12) *S. chilense* (LA1932), (13) *S. huaylasense* (LA1982), (14) *S. arcanum* (LA2150), (15) *S. arcanum* (LA2157), (16) *S. chmielewskii* (LA2663), (17) *S. chilense* (LA2884), and (18) *S. chilense* (LA2930). Mean ± SD labelled with different letters differ significantly at Student’s t-test α = 0.05; n = 3.

To identify tomato lines with resistance against TPP and Lso, we selected a core collection of tomato accessions from 11 wild relative species of cultivated tomato representing the broad diversity of the genus (18 accessions in total). For rapid screening, we performed a no-choice assay using one-month-old tomato plants to evaluate insect survival. Each plant was infested with four adults (two males and two females) carrying Lso haplotype B using clip cages, and adult survival was measured after 10 days (Fig. 1). Accessions were considered to represent potential sources of resistance for breeding purposes when the rate of adult survival was <10%. Resistant accessions with 100% adult mortality included LA0716 (*S. pennellii*), LA1028 (*S. chmielewskii*), LA1777 (*S. habrochaites*), and LA1982 (*S. huaylasense*), while accessions LA0528 (*S. galapagense*) and LA1293 (*S. corneliomulleri*) showed a survival rate of 5.6%. We detected differences in the resistance and susceptibility levels of accessions of the same species, including *S. habrochaites* LA1777 (survival 0%) vs. LA1223 (survival 27.8%) and *S. pennellii* LA0716 (0% survival) vs. LA1926 (24.4% survival), indicating that pest–host interactions are accession-specific rather than widely present in each genus. Therefore, it is possible that additional resistance sources are available in other accessions within the same or different wild species that were not identified in the present study.

In addition to insect resistance, we indirectly measured Lso resistance by detecting the presence of the bacterium at 3 and 5 weeks post inoculation by PCR (Table 1). Lso was detected in distal tissue in 12 out of 18 accessions at 3 weeks post inoculation, and it was detected in three additional accessions (for a total of 15) at 5 weeks post inoculation, indicating that Lso was successfully established and translocated through the vascular tissue of these accessions. By contrast, no Lso PCR amplicons were detected in accessions LA0716 and LA1293 from *S. pennellii* and *S. corneliomulleri*, respectively; these accessions were therefore considered to represent potential sources of resistance. Lso was detected in one *S. chmielewskii* accession LA2663 plant at 3 weeks post inoculation, but no amplicon was detected at 5 weeks post inoculation. Since our method only measured the presence of bacterial DNA in the host, the lack of an amplicon at 5 weeks post inoculation suggests that LA2663 exhibits a late resistance reaction. Alternatively, it is possible that the amplicon detected at 3 weeks post inoculation had been amplified from dead bacteria in the host. However, the fact that Lso DNA was detected in distal tissue indicates effective Lso transmission and translocation in LA2663.

**Table 1:** Lso on wild-type tomato relatives after inoculation, as detected by PCR. The “+” or “−” symbols represent the presence or absence of the OA2/OI2c Lso amplicon per individual replicated plant at the specified time point after inoculation.

Accession	Species	Lso Week 3	Lso Week 5
LA1037	*S. cheesmaniae*	+ + +	+ + +
LA2157	*S. arcanum*	+ + +	+ + +
LA1028	*S. chmielewskii*	+ −	+ +
LA1982	*S. huaylasense*	− − −	+ − +
LA0528	*S. galapagense*	+ − +	− − +
LA1777	*S. habrochaites*	− − +	− − +
LA2930	*S. chilense*	− + +	−+ −
LA1932	*S. chilense*	− − −	+ − −
LA2663	*S. chmielewskii*	+ − −	− − −
LA2150	*S. arcanum*	+ − +	+ − +
LA0716	*S. pennellii*	− − −	− − −
LA0722	*S. pimpinellifolium*	+ − +	− − +
LA2884	*S. chilense*	− − −	+ − −
LA1926	*S. pennellii*	− − +	+ − +
LA1293	*S. corneliomulleri*	− − −	− − −
LA1274	*S. corneliomulleri*	− − −	− −+
LA1223	*S. habrochaites*	+ − −	+ − −
LA1326	*S. neorickii*	− + +	−+ −

In two screenings, *S. pennellii* accession LA0716 and *S. corneliomulleri* accession LA1293 showed resistance to both the vector and pathogen. However, further evaluations are needed to rule out the possibility that the lack of detection of the pathogen resulted from failed transmission by the vector rather than an incompatible host–pathogen interaction.

### TPP has low survival and fecundity in *S. habrochaites* accession LA1777

**Figure 2:**
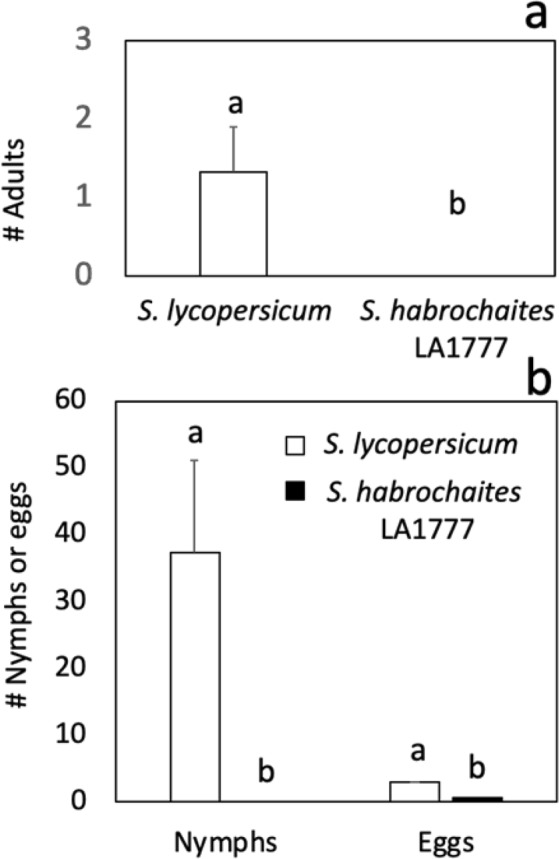
Tomato-potato psyllid survival and fecundity on *S. habrochaites* accession LA1777. Number of (**a**) alive adults and (**b**) nymphs and eggs at 10 days after infestation. Means ± SE labelled with different letters differ significantly at Student’s t-test α = 0.05; n = 6.

We selected *S. habrochaites* for further analysis since it was previously suggested to be a potential source of TPP resistance^30^. We performed a no-choice assay using clip cages to corroborate and expand the results of our rapid screening (Fig. 1). We inoculated one-month-old plants with four young adults (two males and two females) carrying Lso haplotype B and evaluated TPP survival and fecundity. No live adults were found in LA1777-infested plants, representing a 100% reduction in insect survival compared to the susceptible control at 10 days post inoculation. Fecundity was also drastically reduced in LA1777; no live nymphs (100% reduction) were observed, and only one egg (73% reduction) was found compared to the susceptible control (Fig. 2). These observations are in agreement with those of Levy and Tamborindeguy (2014), who detected shorter adult and nymph life spans after inoculation on *S. habrochaites*. The authors also observed reduced host preference compared to the susceptible control, indicating that *S. habrochaites* has both repellent and toxic properties against TPP.

### TPP resistance in *S. habrochaites* is polygenic

To bypass the genetic barriers to hybridisation between wild resistant and cultivated tomato, we took advantage of publicly available mapped recombinant inbred lines (RILs) developed from a cross of *S. habrochaites* (formerly known as *Lycopersicon hirsutum*) accession LA1777 with cultivated tomato (*S. lycopersicum*)^31^. The RILs cover 85% of the *S. habrochaites* genome, with an average introgression contribution of 8.8% per line. These lines were developed as a mapping population to quickly map chromosome regions responsible for traits of interest in LA1777. A detailed genetic map of the *S. habrochaites* RILs is presented in Monforte and Tanksley (2000)^31^.

**Figure 3:**
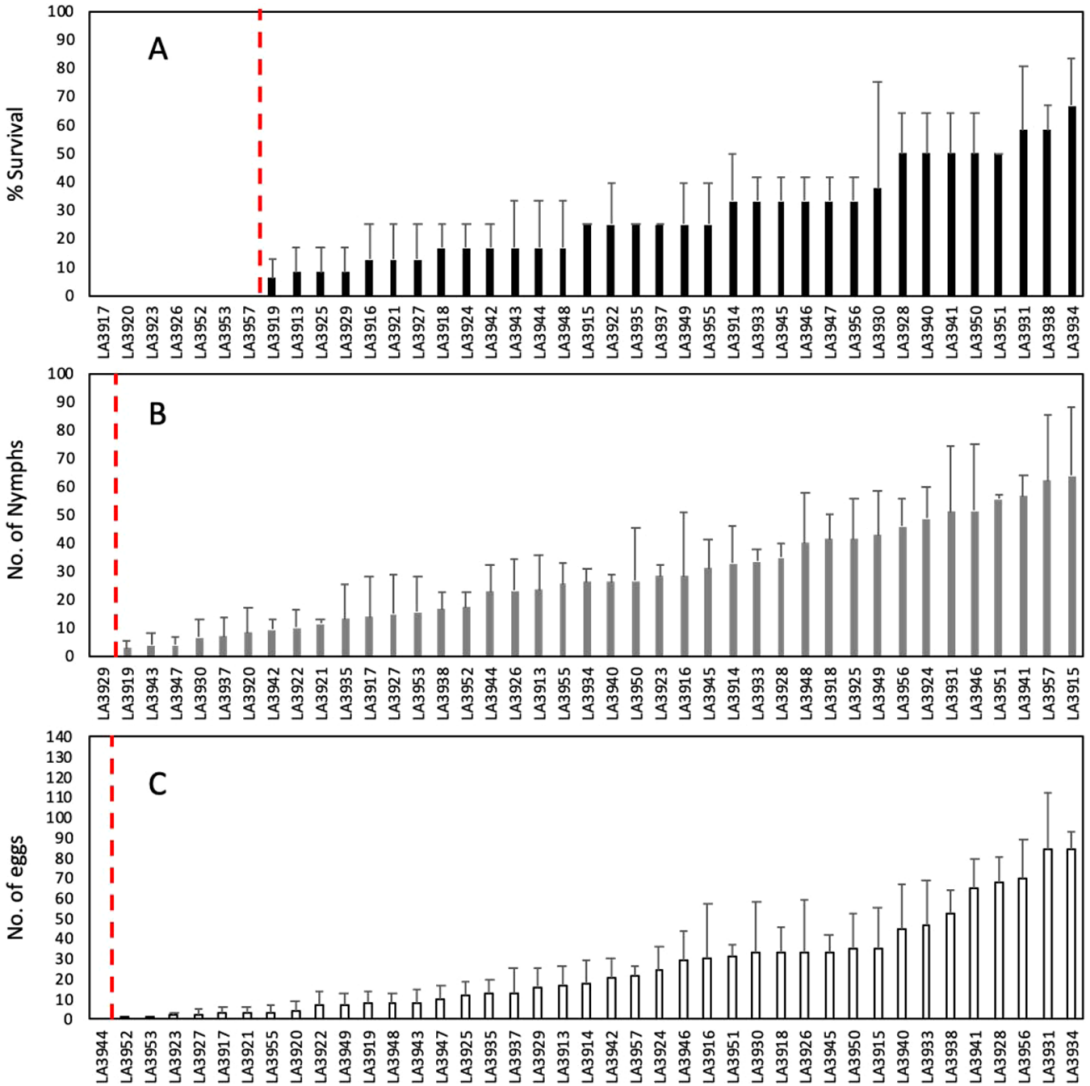
Tomato-potato psyllid survival and fecundity in *S. habrochaites* recombinant inbred lines. **(A**) Adult survival and total number of (**B**) nymphs and (**C**) eggs (as a measure of fecundity) at 10 days after inoculation. Red dashed lines indicate values found in the resistance source *S. habrochaites* LA777. Means ± SE, n = 3–4.

We obtained the core set of RILs from the Tomato Genetics Resource Center at the University of California, Davis for TPP mortality and fecundity evaluations. We performed a no-choice bioassay using four young adult TPP (two males and two females) confined to clip cages for 10 days to measure survival and fecundity. Adult mortality as well as fecundity (number of eggs and nymphs) exhibited a continuous distribution among the RILs, where most lines were susceptible. Compared to the resistant donor parent LA1777, no single RIL showed the same resistance level. However, we detected major QTLs that substantially reduced insect fecundity and survival in some RILs (Fig. 3). For example, no adult survival was observed for seven RILs (LA3917, LA3920, LA3923, LA3926, LA3952, LA3953, and LA3957) (Fig. 3A). As mapped by Monforte and Tanksley (2000)^31^, non-overlapping *S. habrochaites* genome introgressions are present on chromosome 1 (LA3917 and LA3920), chromosome 2 (LA3923), and chromosome 3 (LA3926), while LA3952 and LA3953 contain overlapping introgressions on chromosome 8. Similar resistance levels conferred by the smaller *S. habrochaites* insert in LA3952 spanned by marker TG330 completely overlap with a larger LA3953 fragment (markers TG330, CT265, CT68, and TG480)^31^, indicating that the resistance factor is present in the shorter insert in LA3952. Therefore, we considered shorter introgression in LA3952 as the one carrying major QTL for subsequent analysis for a total of six major QTLs in the same number of accessions.

In addition, major QTLs associated with substantial reductions in fecundity were detected in the RILs (Fig. 3B,C). For example, RILs LA3929 (harbouring an insertion in chromosome 3) did not show any live nymphs, and no eggs were found in RIL LA3944 (harbouring an introgression on chromosome 6). Together, these results indicate that resistance in LA1777 is controlled by more than one gene, since introgressions on chromosomes 1, 3, 5, 6, and 8 contribute to TPP resistance in *S. habrochaites*. Furthermore, individual chromosome insertions appear to control different aspects of resistance (i.e., adult mortality vs. fecundity), although some overlap might occur. For example, QTL in RIL LA3952 was associated with high adult mortality and very few eggs, suggesting that the insertion in chromosome 8 is responsible for different aspects of resistance, although we cannot rule out the possibility that the low numbers of eggs are an effect of rapid mortality. Individual QTLs with strong effects found in single RILs can provide enough protection against insects and could therefore be used for breeding purposes. For example, the identified RILs with major QTLs for TPP resistance on chromosomes 1, 3, 5, 6, and 8 could be crossed with tomato breeding lines for cultivar development.

Similar to our findings, resistance traits to chewing and phloem-feeding insects introgressed from wild-tomato relatives have previously shown multigenic inheritance. For example, resistance introgressions on chromosomes 1, 8, and 10 from *S. pennellii* and 6 and 8 from *S. galapagense* against Colorado potato beetle (*Leptinotarsa decemlineata*) harbour QTLs associated with significant reductions in leaf damage and larval growth^32^. Likewise, major QTLs conferring resistance to sweet potato whitefly (*Bemisia tabaci*) on chromosome 9, 10, and 11 affecting oviposition have been reported^33^; these major QTLs are associated with a higher density of type IV trichomes.

### Plant defence hormones are required for basal resistance against TPP in susceptible plants

Plants protect themselves from pests and diseases via different mechanisms^34^. In some plant–pest interactions, resistance is mediated by a single dominant resistance gene (so-called *R*-gene)^27^. However, even without the presence of *R*-genes, plants can deploy defence mechanisms that reduce the extent of damage from insect infestation. These defences, described as basal, as well as *R*-gene-mediated defences, are dependent on the interactions of multiple genes that elicit constitutive and induced responses, including hormone and transcriptional reprogramming^35–37^.

**Figure 4:**
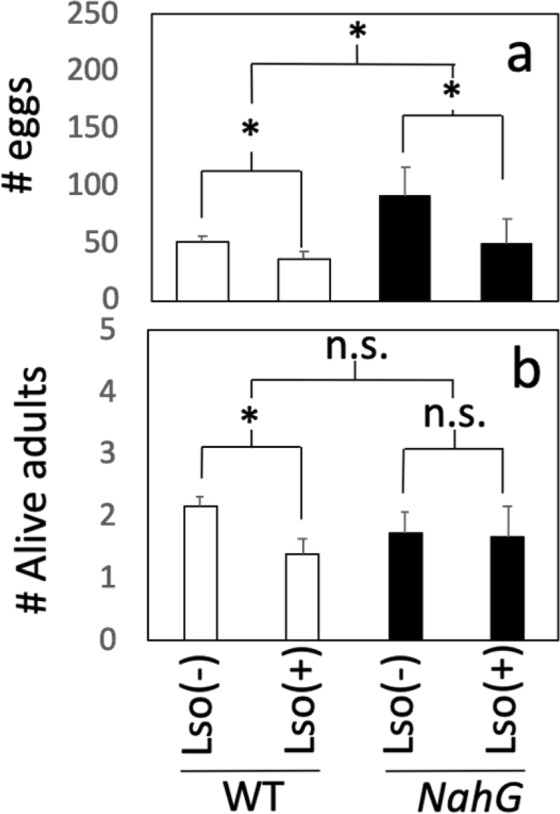
Tomato-potato psyllid survival and fecundity on salicylic acid-deficient plants at 10 days after infestation (dai). Insect (**a)** survival and (**b)** fecundity in wild-type tomato cv. MoneyMaker and salicylic acid-deficient transgenic *NahG* plants. Lso (+) and Lso (−) indicate the presence or absence of *Candidatus* Liberibacter solanacearum in adult tomato-potato psyllids. Means ± SE labelled with an asterisk (*) differ significantly at Student’s t-test α = 0.05; n = 6.

To determine the role of the plant defence hormone SA on basal defence signalling against TPP, we used a transgenic line carrying the bacterial *NahG* gene encoding salicylate hydroxylase, which degrades SA to catechol^38^. Transgenic plants expressing *NahG* exhibit reduced SA accumulation, in turn reducing SA-dependent defence responses^38^. We performed a no-choice bioassay showing that SA deficiency resulted in a significant increase in the number of eggs in inoculated plants 10 days after (Fig. 4a) but had no significant effect on adult mortality (Fig. 4b). These findings indicate that SA participates in basal resistance against TPP, as intact SA signalling is required to reduce insect fecundity.

Although no major effect of SA on adult TPP survival was observed, we reasoned that SA might participate in the Lso–tomato–TPP interaction, since its deficiency eliminates differences in adult survival between TPP Lso-carrying vs. Lso-free insects. Similar to previous observations^39^, we determined that TPP carrying Lso are less fit than Lso-free insects. While these differences in adult survival and fecundity between TPP with or without Lso were observed in the tomato with intact SA signalling (Fig. 4a,b), we detected no significant difference in TPP survival between TPP Lso positive and negative insects in tthe *NahG* SA-deficient transgenic line (Fig. 4b). Therefore, perhaps Lso modulates adult survival directly, by interacting with the vector (i.e., as an insect pathogen) or indirectly, by eliciting broad defences in the plant host. For example, the presence of the plant-associated bacterium *Pseudomonas syringae* decreases survival but increases oviposition of pea aphid (*Acyrthosiphon pisum*)^40^.

### TPP elicits local SA signalling in *S. habrochaites* RIL LA3952 independently of Lso

In addition of the role of SA in basal resistance, we evaluated the role of this plant hormone in *S. habrochaites* RIL LA3952 by measuring the expression of the *pathogenesis-related gene P4* in tomato (GenBank identifier 544185) in locally infested foliage by RT-qPCR. The *P4* gene is homologous to *PR1a* in tobacco (*Nicotiana tabacum*) and *Arabidopsis thaliana* and is upregulated in response to exogenous SA application^41–43^.

**Figure 5:**
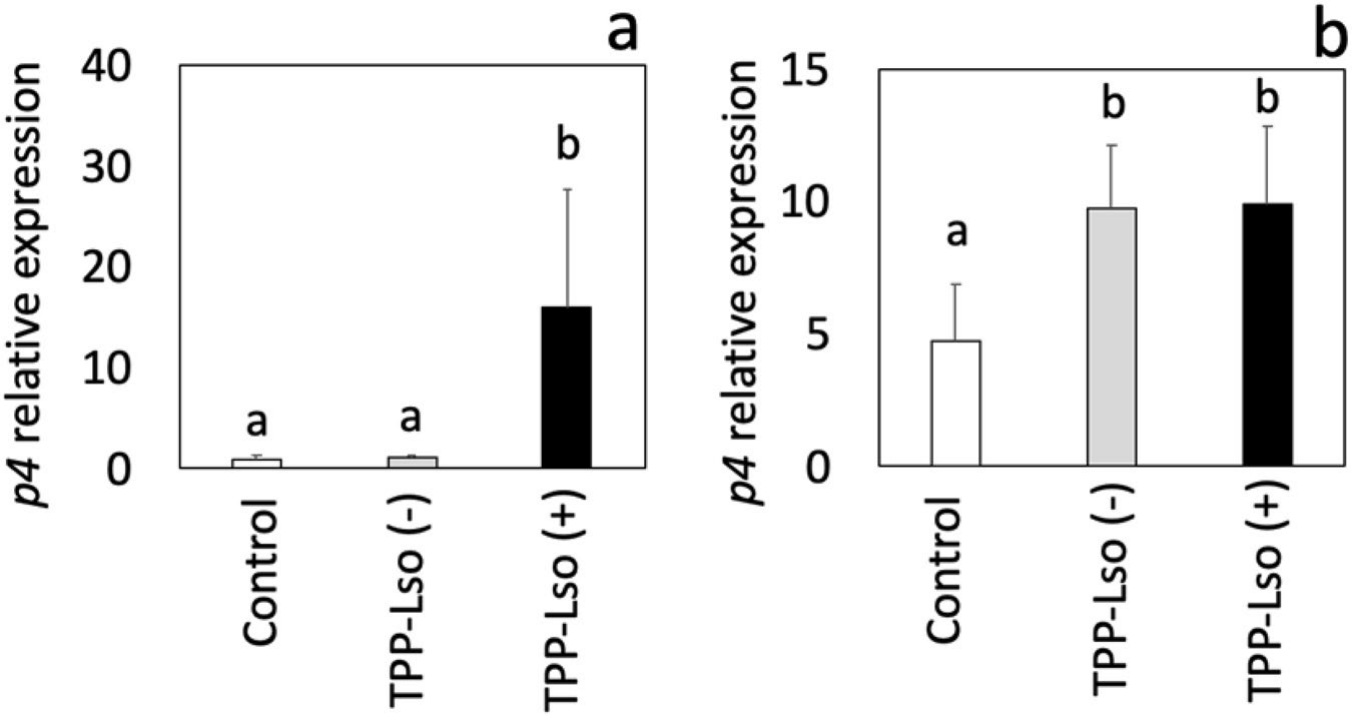
Intudent’s t-test α = 0.05; n = 6.duction of salicylic acid signaling in response to TPP-Lso infestation. Expression of *pathogenesis-related 4* (*P4*) 48 hours after infestation in (**a**) susceptible *S. lycopersicum* and (**b**) resistant *S. habrochaites* recombinant inbred line LA3952. Lso (+) and Lso (−) indicate the presence or absence of *Candidatus* Liberibacter solanacearum in adult tomato-potato psyllids. Means ± SE labelled with different letters differ significantly at Student’s t-test α = 0.05; n = 4.

We infested tomato plants with 25 adult Lso-free or Lso-containing TPP, confined them in sleeve cages for 48 h, and collected locally infested tissue to measure the expression of the SA-signalling marker gene *P4*. In susceptible plants, Lso-free insects did not induce SA signalling alone (Fig. 5a), indicating that SA-mediated defence signalling is elicited in plants in response to bacteria but not TPP infection. These results suggest that the reduced TPP fitness at least partially resulted from an indirect effect of Lso on plant defence induction. This notion is in agreement with the previous finding that TPP induces SA signalling and that the level of induction parallels with the bacterial titre in the insect^44^. However, the same authors reported that in the absence of the psyllid, Lso suppressed plant defence responses, including the expression of SA-responsive genes in tomato, suggesting that Lso might manipulate plant defence responses to benefit its vector rather than reduce its fitness. However, since their analysis was performed by grafting Lso-infected plants to transmit the bacteria into healthy plants due to the unavailability of Lso-free psyllids, their observations might reflect systemic suppression of defence signalling rather than the effect of local induction, as detected in the current study.

Contrary to what was observed in susceptible plants, in the resistant RIL LA3952, the presence of TPP upregulated the SA marker gene independently of Lso (Fig. 5b), and LA3952 plants showed constitutively higher transcript levels in the non-inoculated control compared to *S. lycopersicum*, pointing to the involvement of this hormone in *S. habrochaites* resistance.

SA plays an active role in defence signalling in interactions between resistant tomato and other phloem-feeding insects, suggesting that plants might employ conserved mechanisms against insects with similar feeding behaviours. For example, the loss of function of FATTY ACID DESATURASE7 (FAD7) and the *Mi-1.2* gene in tomato result in lower potato aphid (*Macrosiphum euphorbiae*) fitness, which is associated with enhanced SA signalling, whereas SA suppression results in increased fitness^36,45^. Further functional analyses are needed to validate the role of SA in *S. habrochaites* resistance.

### Resistance in *S. habrochaites* RIL LA3952 requires the presence of Lso to increase psyllid mortality but not to reduce oviposition

Since microbes can positively or negatively modulate interactions between insects and plants^39,46^, the impact of the microbial communities associated with insects and plants on their mutual ecological success should be taken into consideration. An intriguing category of microbial symbionts are those that are fastidious, have reduced genomes, and are found in both insect and plant hosts because these microbes have had to adapt to two different eukaryotic organisms. This group includes some of the most important insect-borne plant pathogens, such as Lso transmitted by TPP and *Candidatus* Liberibacter asiaticus (Lsa) carried by the citrus psyllid *Diaphorina citri*^47,48^. Therefore, it is important to study three-way interactions between the vector, pathogen, and host plant to understand the dialog among partners.

**Figure 6:**
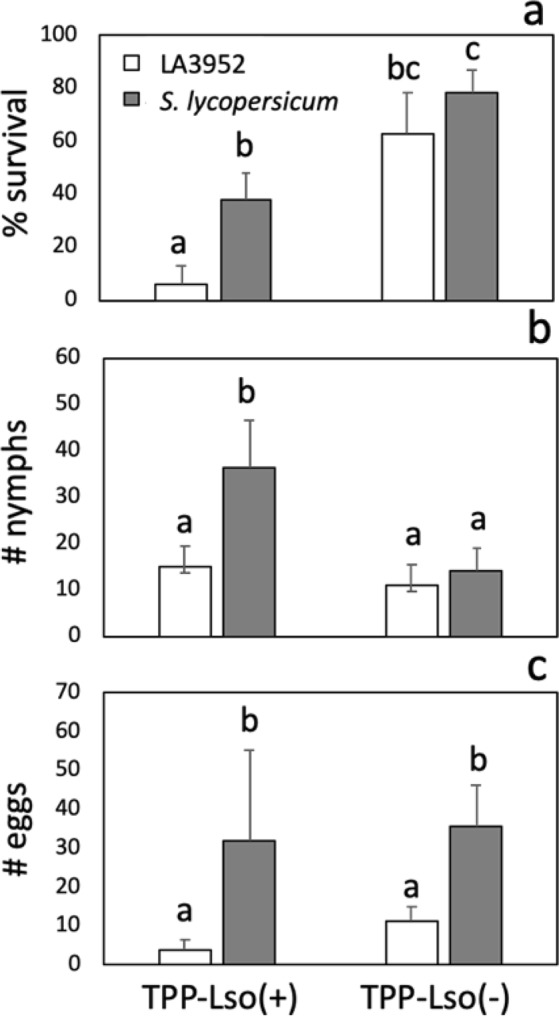
Tomato-potato psyllid (TPP) fitness in the presence (+) and absence (−) of *Candidatus* Liberibacter solanacearum (Lso) on *S. habrochaites* RIL LA3952. Tomato-potato psyllid (**a)** survival and (**b**,**c**) fecundity 10 days after infestation. Means ± SE labelled with different letters differ significantly at Student’s t-test α = 0.05; n = 8.

We observed that the *S. habrochaites* introgression in RIL LA3952 contributes to resistance by reducing adult TPP survival and fecundity (Fig. 3). However, our evaluations were performed with insects carrying the plant pathogenic bacterium Lso haplotype B. Nevertheless, it is unknown if Lso tunes resistance in LA3952 since, in susceptible tomato, the presence of the bacterium resulted in lower insect fitness. To address this question, we evaluated insect fitness in resistant LA3952 plants inoculated with Lso-free TPP. In a no-choice assay, adult and nymph mortality were not significantly reduced in Lso-free insects, but the number of eggs was significantly reduced, indicating that the effect of LA3952 on insect oviposition is independent of Lso (Fig. 6).

### Resistance in *S. habrochaites* is complex and may involve different defence mechanisms

This study identified several *S. habrochaites* genome introgressions in tomato that participate in overlapping or exclusive aspects of TPP survival and fecundity. Furthermore, we showed that the presence of the plant pathogen, Lso, carried by TPP influences the onset of resistance.

Levy and Tamborindeguy (2014) showed that *S. habrochaites* not only influences insect fitness by reducing longevity and fecundity, but also reduces host preference. Analysis of volatile emissions of *S. habrochaites* (PI127826) revealed the presence of curcumene, zingiberene, terpinene, and caryophyllene^49^, which might have toxic or repellent effects on insect vectors. Moreover, sesquiterpenes with potential insecticidal properties have been identified in *S. habrochaites* RILs with insertions in chromosome 6 and 8^50^.

In addition to the effects of plant genotype on tomato–TPP interactions, the effects of differences in bacterial and insect populations must be considered. For example, disease symptoms and Lso abundance in infected tomato plants vary depending on the Lso haplotype (A or B)^51^. Plants infected with haplotype B had more severe symptoms (resulting in earlier plant death) than haplotype A-inoculated plants. With the discovery of haplotype F in potato plants^52^, additional analyses are needed to determine its effect on tomato–psyllid interactions. Similarly, at least four TPP biotypes have been reported in the United States, including the central (Texas and Nebraska), southwestern (Arizona), western (California and Washington), and northwestern (arisen from the western biotype)^2,53^. Further analyses should be performed to determine if resistance differs among biotypes.

Since our initial functional analyses were limited to a single QTL in RIL LA3952, the molecular mechanisms underlying resistance in the remaining RILs remain unknown, including that of the original resource, *S. habrochaites* accession LA1777. Further studies involving various pathogens and vectors are needed to completely characterise the resistance mechanisms associated with the major QTLs.

### *S. habrochaites* RILs could be utilised in tomato breeding programs for cultivar development

Although no single *S. habrochaites* RIL showed the same level of resistance as the original LA1777 donor, major QTLs with high resistance levels were detected in some introgression lines, which therefore could potentially be used in breeding programs to enhance resistance in elite tomato breeding lines. However, in addition to the complex inheritance and resistance mechanisms of these traits, the utilisation of exotic germplasm resources depends on the production of a fertile hybrid and genetic recombination between the donor and recipient chromosomes^11^. While the use of RILs has already allowed us to bypass the first constraint, it is unknown if undesirable traits (linkage drag) are also present in the large insertions of the *S. habrochaites* genome and if recombination in insertion loci would be sufficient to select against these traits. There are several successful examples of the use of *S. habrochaites* as a source of new traits, including chilling tolerance^17^, resistance to shoot wilting^54^ and TYLCV^19^, indicating that it is feasible to overcome these barriers. Regardless of these potential genetic obstacles, the advantage of using the newly identified RILs with major TPP resistance QTLs described in this study is that the initial hybridisation barriers and low fertility associated with interspecific crosses have already been bypassed, making them highly amenable for use in conventional breeding programs.

## Methods

### Plant materials

Wild tomato relative accessions and *S. habrochaites* recombinant inbred lines were obtained from the Tomato Genetic Resource Center (TGRC) at the University of California, Davis, CA. The TGRC accession numbers are listed in Table 1. Cultivated tomato (*S. lycopersicum* cv. MoneyMaker) and *NahG* transgenic seeds in the cv. MoneyMaker background were kindly donated by Fiona Goggin, University of Arkansas, Fayetteville, AR.

### Insect colonies

Lso-free TPP colonies and TPP colonies carrying Lso haplotype B of the Western biotype were reared in confining cages containing tomato and pepper plants. Old plants were replaced by pathogen- and pest-free plants as needed to maintain the insect populations. The colonies were periodically tested for Lso (including before each bioassay) by PCR using primer set OA2 forward 5′-GCGCTTATTTTTAATAGGAGCGGC-3′^4^ and OI2c reverse 5′-GCCTCGCGACTTCGCAACCCAT-3′^55^ targeting the 16S rRNA gene of Lso to detect its presence. Amplifications were performed in 25 µL reactions with Green GoTaq Flexi Polymerase (Promega, Madison, WI) following the manufacturer’s protocol. For each reaction, 1 µl (5 µM) of each primer and 2 µL of DNA extract (~40 ng) were incubated at 95 °C/5-min for initial denaturation, 95 °C/30-sec, 65 °C/30-sec, and 1 min at 72 °C/1-min for 40 cycles, followed by a final 10-min incubation at 72 °C. The PCR products were separated on 1.5% agarose gels containing Gel Red^®^ (Biotium, Fremont, CA). The Lso haplotype was also tested by PCR using SSR primer pairs Lso-SSR-1F forward 5′-TTATTTTGAGATGGTTTGTTAAATG-3′ and Lso-SSR-1R reverse 5′-TATTATCATTCTATTGCCTATTTCG-3′^56^ via incubation at 95 °C/6-min for initial denaturation, 95 °C/30-sec, 58 °C/30-sec, and 1 min at 72 °C/1-min for 35 cycles, followed by a final 7-min incubation at 72 °C. The PCR products were separated on 1.5% agarose gels containing Gel Red^®^ (Biotium, Fremont, CA) (data not shown).

### Insect no-choice bioassays

Insect survival and fecundity (number of eggs and nymphs) were measured 10 days after placing two young male and two young female Lso-free or Lso-haplotype B-carrying TPP on the terminal leaflet of the second fully opened leaf from the apical meristem. The psyllids were confined using clip cages supported with floral wire to avoid wounding. For population bioassays, 3–4 replications (e.g. number of plants) were performed while 6–8 replications were performed in accession specific bioassays (see figure legends for details) in a complete randomized design. Plants were kept at 23 °C with 14 hrs light and at 10 days post infestation, the live adults, nymphs, and eggs were counted using an Olympus SZ60 dissecting microscope (Center Valley, PA). Data were analysed by ANOVA, and means for significant effects at α = 0.05 were separated using Student’s *t*-test with JMP version 14.0 (SAS Institute, Cary, NC).

### Lso detection by PCR

Three individual plants per accession were inoculated with 10 adult TPP carrying the Lso haplotype. Systemic tissue was collected at 3 and 5 weeks after infestation from the top fully opened leaf from the apical meristem as an indicator of vascular pathogen translocation and establishment. DNA was extracted from each sample as described below, and PCR was performed using primer pairs OA2/OI2c as described above. The *RPL2* primer set was used as a PCR amplification control for all samples.

### DNA isolation

Plant genomic DNA was extracted from ~200–500 mg of fresh leaf tissue using the cetyltrimethylammonium bromide (CTAB) method described by Hoisington *et al*. (1994)^57^. Insect DNA was extracted by macerating a single TPP in CTAB with a micropestle as described by Sengoda *et al*. (2014)^58^. DNA concentration and purity were estimated using a NanoDrop spectrophotometer (Thermo Fisher Scientific, Waltham, MA).

### RNA isolation and cDNA synthesis

Local leaf tissue samples were carefully collected from each plant, immediately flash frozen in liquid nitrogen, and stored at −80 °C until extraction. Total RNA was extracted using TRIzol reagent (Invitrogen, Carlsbad, CA) following the manufacturer’s protocol. After extraction, the RNA was treated with TURBO DNA-free (Ambion, Foster City, CA) DNase. cDNA synthesis was performed using iScript^TM^ reverse transcription SuperMix (Bio-Rad, Hercules, CA) using 1 µg of DNase-treated RNA in a 20-µL reaction volume following the manufacturer’s protocol.

### Gene expression analysis

Cultivated tomato *S. lycopersicum* cv. MoneyMaker and *S. habrochaites* RIL LA3952 plants were inoculated with 25 adult Lso-free or Lso-haplotype B-carrying TPP using one-month-old plants by confining a single leaf of each plant in an organza bag cage. Empty cages were used as a no-infestation control and four replications per treatment were performed. Local tissue was collected 48 h after infestation to measure the expression of the SA-induced plant defence signalling marker gene *PATHOGENESIS-RELATED4* (*P4*). RNA extraction and cDNA synthesis were performed as described above. The cDNA was diluted to 40 µL, and a 2 µL aliquot equivalent to 25 ng RNA was used as template. Two technical replicates per biological and no-template controls were included in the quantitative RT-PCR experiments. The qPCR was performed in a CFX384 Touch^TM^ real-time PCR detection system (Bio-Rad, Hercules, CA) using Universal SYBR^®^ Green Master Mix (Bio-Rad, Hercules, CA) scaled for a 20 µL reaction volume, with final primer concentrations of 0.5 mM. The PCR conditions were as follows: 15 min of activation at 95 °C; 40 amplification cycles (denaturation at 94 °C for 15 s, annealing at 59 °C for 30 s, and extension at 72 °C for 30 s); and a final data acquisition step to generate melting curves (95 °C for 1 min and 55 °C for 30 s). Dissociation plots were examined for the presence of a single amplicon per primer set. The primer pairs used for RT-qPCR were as follows: *P4* (GenBank accession no. M69247 for mRNA sequence) forward (5′-CAACTCAAGAGCGGGTAGTTG-3′) and reverse (5′-CCACACATTTTTCCACCAACAC-3′) and *RPL2* (GenBank accession no. X64562) forward (5′-GAGGGCGTACTGAGAAACCA-3′) and reverse (5′-CTTTTGTCCAGGAGGTGCAT-3′). Primer efficiency was calculated by performing serial dilutions of a pooled set of cDNA from all samples using the E = 10^[−1/Ct slope]^ method^59^. Relative gene expression levels were adjusted by primer efficiency and normalised to the expression of the endogenous gene *RPL2* as described by Pfaffl (2001)^60^. Gene expression levels for each treatment group were calculated relative to the susceptible empty cage control. The relative expression values for each treatment were log_2_ transformed to stabilise variances. Data were analysed by one- or two-way ANOVA, and means for significant effects at α = 0.05 were separated using Student’s *t*-test with JMP version 14.0 (SAS Institute, Cary, NC).
